# Hospital-to-home transitions for children with medical complexity: part 2—a core outcome set

**DOI:** 10.1007/s00431-023-05049-2

**Published:** 2023-06-20

**Authors:** Heleen N. Haspels, Annemieke A. de Lange, Mattijs W. Alsem, Bettina Sandbergen, Karolijn Dulfer, Matthijs de Hoog, Koen F. M. Joosten, Clara D. van Karnebeek, Job B. M. van Woensel, Jolanda M. Maaskant

**Affiliations:** 1 Department of Pediatric Intensive Care Unit, Amsterdam Reproduction and Development, Amsterdam UMC location University of Amsterdam, Emma Children’s Hospital, Meibergdreef 9, Amsterdam, The Netherlands; 2 Department of Pediatric and Neonatal Intensive Care, Division of Paediatric Intensive Care, Erasmus MC-Sophia Children’s Hospital, Rotterdam, 3015 CN The Netherlands; 3 Department of Pediatrics, Amsterdam UMC location University of Amsterdam, Emma Children’s Hospital, Meibergdreef 9, Amsterdam, 1105 AZ The Netherlands; 4 Department of Rehabilitation, Amsterdam Movement Sciences, Amsterdam UMC location University of Amsterdam, Meibergdreef 9, Amsterdam, The Netherlands; 5 Expert by experience, Amsterdam, The Netherlands; 6 Department of Pediatrics and Human Genetics, Emma Center for Personalized Medicine, Amsterdam Reproduction and Development, Amsterdam UMC location University of Amsterdam, Emma Children’s Hospital, Meibergdreef 9, Amsterdam, The Netherlands

**Keywords:** Transitional care, Children with Medical Complexity, Core Outcome Set, Delphi study, Focus groups

## Abstract

Appropriate outcome measures as part of high-quality intervention trials are critical to advancing hospital-to-home transitions for Children with Medical Complexity (CMC). Our aim was to conduct a Delphi study and focus groups to identify a Core Outcome Set (COS) that healthcare professionals and parents consider essential outcomes for future intervention research. The development process consisted of two phases: (1) a three-round Delphi study in which different professionals rated outcomes, previously described in a systematic review, for inclusion in the COS and (2) focus groups with parents of CMC to validate the results of the Delphi study. Forty-five professionals participated in the Delphi study. The response rates were 55%, 57%, and 58% in the three rounds, respectively. In addition to the 24 outcomes from the literature, the participants suggested 12 additional outcomes. The Delphi rounds resulted in the following core outcomes: (1) disease management, (2) child’s quality of life, and (3) impact on the life of families. Two focus groups with seven parents highlighted another core outcome: (4) self-efficacy of parents.

*Conclusion*: An evidence-informed COS has been developed based on consensus among healthcare professionals and parents. These core outcomes could facilitate standard reporting in future CMC hospital to home transition research. This study facilitated the next step of COS development: selecting the appropriate measurement instruments for every outcome.

**Table:** 

**What is Known:** *• Hospital-to-home transition for Children with Medical Complexity is a challenging process.* *• The use of core outcome sets could improve the quality and consistency of research reporting, ultimately leading to better outcomes for children and families.*	
**What is New:** *• The Core Outcome Set for transitional care for Children with Medical Complexity includes four outcomes: disease management, children’s quality of life, impact on the life of families, and self-efficacy of parents.*

**Supplementary Information:**

The online version contains supplementary material available at 10.1007/s00431-023-05049-2.

## Introduction

Children with medical complexity (CMC) comprise a diverse population with chronic conditions, functional limitations, substantial family-identified service needs, and high healthcare use [1]. CMC have a variety of diagnoses such as congenital heart diseases, cerebral palsy, metabolic diseases, neurological conditions, or epilepsy. Although CMC represents a small proportion of the pediatric population [2, 3], their healthcare utilization is substantial due to frequent emergency department visits and often lengthy and complicated (re)hospitalizations [4–6]. CMC are characterized by complex home care since they often require nursing care, medical equipment, advocacy, and frequent inpatient contact with different medical subspecialists [7]. The transition from hospital (where healthcare professionals are responsible for the care) to home (where parents are the most important and responsible caregivers) is a vulnerable process with many challenges and obstacles. Parents have to learn complex nursing care, need support in logistic and coordination issues, and emotional support [8–11].

Therefore, it is of great importance to guide the hospital-to-home transition carefully. Various interventions have been developed to improve this transition, such as transitional care units [12, 13], telemedicine [14, 15], post-discharge nurse home visits [16], and post-discharge caregiver coaching [17]. Research on the effectiveness of interventions to support hospital-to-home transitions is growing [18–21]. Most studies reflected on the life impact (the impact on functioning, quality of life, delivery of care, and personal circumstances) and resource use [22]. The most commonly reported outcomes are the self-efficacy of parents, amount of hospital (re)admissions, and length of stay in the hospital [22]. However, outcomes such as out-of-pocket expenses [23], disease management [14], and adverse events [24] have also been investigated. A core outcome may facilitate the comparison of findings in systematic reviews and meta-analyses, which rely on consistent outcome reporting to pool data across studies. To move forward in this field there is a need for consensus on a Core Outcome Set (COS) to systematically develop and evaluate hospital-to-home interventions.

A COS is “an agreed minimum set of outcomes that should be measured and reported in all clinical trials of a specific health condition, trial population, and/or intervention” [25]. A COS is considered as a fundamental list of outcomes and is not meant to restrict researchers from measuring additional outcomes relevant to a specific study [26]. To date, no COS for transitional care of CMC exists. Therefore, building on a previously created overview of outcomes reported in the literature [22], this study aimed to reach a consensus on a set of core outcomes informed by healthcare professionals and parents.

## Methods

We executed a three-round Delphi study followed by focus group discussions. The study followed the COMET methodological recommendations [25] and registered the protocol on the COMET database (www.comet-initiative.org/Studies/Details/1899) [27]. The study is reported in line with COS-Standards for Reporting (COS-STAR) guidance [28] and the GRIPP2 short form for Patient and Public Involvement (PPI) [29].

### Research ethics

Approval was provided by the Institutional Review Board of the Amsterdam UMC, location AMC, who waived the need for a full ethical review (W20_220#20.007). Participants for the Delphi survey were informed and asked for consent during recruitment and once more in the introduction of the Delphi survey. Parental informed consent was obtained during the focus group discussions.

### Parent involvement

Our study involved parents at three distinct levels to ensure the inclusion of the parents’ perspective in the COS. Firstly, a parent caring for a child with medical complexity and active as a representative of a patient organization (B. S.) was part of our research group. The parent representative provided valuable input throughout the study and contributed to the protocol development by advising how to include parents’ perspectives best. Besides participating in research meetings, the parent representative participated in the pilot testing of the questionnaires for the Delphi study. Additionally, questionnaires for the focus groups were pilot tested to make sure the information letter and questions asked were understandable for other parents. Secondly, representatives of parents’ interest groups were invited to participate in the Delphi survey. Finally, we organized a series of focus groups specifically for parents, which allowed them to share their perspectives and experiences related to the research topic.

### Delphi study

#### Participants

Participants were identified through the literature review [22] and recommendations from recognized professionals in the field. Since CMC deal with many healthcare professionals, we aimed to include opinions from different disciplines. Therefore, we selected professionals from different disciplines, e.g., pediatricians, pediatric intensivists, neonatologists, pediatric nurses, specialized pediatric nurses (e.g., nurse practitioner), psychosocial care workers, and allied healthcare professionals, who all were involved in the hospital-to-home transition of CMC. This resulted in a purposive sample of participants, homogeneous in the field of transitional care, but heterogeneous in terms of professional background. To guarantee the perspectives of patients, also representatives of parents’ interest groups participated in the same manner as the professionals. Parents were recruited through our Transitional Care Unit (TCU) Consortium [12]. This consortium is established in the Netherlands to develop a standard of care for the hospital-to-home transition for CMC.

#### Questionnaires

Questions for the Delphi rounds were based on the preliminary results of our systematic review, where we identified 24 unique outcomes [22]. These outcomes were categorized into domains according to the taxonomy of Dodd et al. [30]. Links to references were given for every outcome. The questionnaires were distributed online through a web application (Castor EDC). Participants were asked to rate each outcome for inclusion in the COS using a five-point Likert scale (strongly disagree, disagree, neutral, agree, strongly agree). Six potential participants (two medical doctors, three nurses, and one researcher) piloted the first questionnaire to assure clarity. It was tested if Castor EDC worked properly, if the questions and answers options were clear, and how long it took to complete the questionnaire. No adjustments were made after the pilot test. The data from the pilot test have not been used in the analysis. The Delphi rounds took place between October 2021 and February 2022. Professionals were given 4 weeks to complete each round. Non-responders received two motivational e-mails after 2 and 4 weeks, and deadlines were communicated to prevent attrition [31]. At least two of the three questionnaires must have been filled in by the same participant to be included in the analyses. Participants who filled in only one of the three Delphi rounds were excluded from the analyses.

#### Delphi rounds

The questionnaire in round 1 consisted of three parts. Firstly, demographic information of the participants was collected. Secondly, the 24 outcomes were presented according to the aforementioned structure. Thirdly, respondents could propose novel outcomes and add free-text comments. The research group reviewed new suggestions to ensure they represented a new outcome.

The following definitions were used for the consensus threshold: when at least 70% of the professionals rated an outcome with “agree” or “strongly agree,” consensus for inclusion in the COS was reached [25, 32]. Consensus for exclusion in the COS was reached if at least 70% of the professionals rated the corresponding outcome as “disagree” or “strongly disagree”. The outcomes on which consensus was reached were not presented for re-scoring in the next round.

In round 2, the remaining outcomes were listed with the group response results, described as percentages of the professionals’ scores on the 5-point Likert scale. Professionals were asked to re-score these outcomes, as well as the newly suggested outcomes. Again, outcomes that reached consensus based on the cutoff value of 70% were excluded from round 3.

Round 3 consisted of two parts. In the first part, the outcomes that had not reached consensus in the previous rounds were shown, again with the results in percentages. Professionals were asked to re-score the outcomes. The second part was added since, during the previous two Delphi rounds, participants highlighted that it was hard to fill out the questions since all outcomes were considered important in transitional care. Therefore, we presented the outcomes that reached consensus for inclusion in de COS so far, and asked to confirm if they considered the outcome a *core* outcome (mandatory in all clinical trials regardless the objective of the study) or an *important* outcome (relevant, but only when related to the aim of a specific study). It was stressed that the *core* outcomes should be limited to outcomes relevant in *all* studies.

### Focus groups

Semi-structured online focus groups with parents of CMC were organized to validate the results of the Delphi study and to identify missing outcomes important from the parents’ perspective. Twenty-seven parents in the Netherlands who were (1) Dutch-speaking, (2) > 18 years old, and (3) had experience with the hospital-to-home transition were invited to participate. Parents were recruited through the TCU Consortium [12] and the parent representative in our research group (B. S.). Purposeful sampling was used to maximize diversity among the recruited parents. This involved identifying and selecting parents of CMC with various underlying diagnoses.

Once parents agreed upon participation in the study, they received an information letter with all outcomes considered in the Delphi rounds. Demographics of parents were collected using a short online questionnaire. H. H. moderated the focus groups with support of a second researcher (J. M. or M. A.) all experienced in conducting qualitative research such as focus groups and semi-structured interviews. After a personal introduction, parents were asked about their experiences regarding the hospital-to-home transition. Thereafter, the mentioned outcomes were discussed as well as any missing outcomes. During the discussion, we explored which outcomes were *core* from the perspective of the parents. Interaction and discussion between the participants were encouraged, but the researchers intervened when the debate strayed away from the purpose of the focus group.

### Analyses

The results of the Delphi questionnaires were summarized as frequencies and percentages. The level of agreement was expressed in percentages of the same answers to each question. Based on the 70% level of agreement, outcomes that reached consensus were considered important outcomes in rounds 1, 2, and 3 (first part). Inclusion in the COS occurred when at least 70% of the participants rated the outcome as *core* in the second part of round 3. Response rates were calculated as the number of respondents who completed a Delphi round as a proportion of the total invited group.

The focus groups were recorded, and three researchers (H. H., J. M., M. A.) replayed and summarized the transcripts independently. The researchers discussed their notes and looked for recurring topics or issues that were discussed by multiple participants to reach a consensus on the core outcomes mentioned by parents. Finally, the results of the focus groups were discussed in the research group until consensus on the final COS was reached.

## Results

### Delphi study

#### Participants

In total, 110 professionals were invited to participate in the Delphi study, of whom 67 consented to participate. A total of 45 out of 67 (67%) filled out at least two questionnaires. Of the 45 participants, most lived in the Netherlands (73.3%), followed by Canada (13.3%) and the USA (13.3%). The sample consisted of different healthcare disciplines and three parent representatives. The characteristics of the respondents are summarized in Table 1.

**Table 1:** Characteristic professional Delphi study (*n* = 45)

**Baseline characteristics**	
Geographic location, *n* (%)	
Canada	6 (13.3)
The Netherlands	33 (73.3)
USA	6 (13.3)
Profession, *n* (%)	
Pediatrician	10 (22.2)
Neonatologist	1 (2.2)
Pediatric intensivist	3 (6.7)
Pediatric nurse	12 (26.7)
Specialized pediatric nurse, e.g., nurse practitioner	8 (17.8)
Allied health care professionals	5 (11.1)
Psychosocial care workers	3 (6.7)
Patient representative	3 (6.7)
Postgraduate experience in years, *n* (%)	
< 10 years	16 (35.6)
11–20 years	11 (24.4)
> 20 years	17 (37.8)
Missing	1 (2.2)
Highest level of education, *n* (%)	
Bachelor’s degree	17 (37.8)
Master’s degree	18 (40)
Post-master’s degree (PhD)	8 (17.8)
Missing	1 (2.2)
Organization, *n* (%)	
Children’s hospital	23 (51.1)
Primary care, home care	11 (24.4)
Patient foundation	3 (6.7)
Other^a^	7 (15.6)
Missing	1 (2.2)

^a^University, School of Nursing, Centre of Pediatric Expertise, Women Hospital

#### Delphi rounds

Of the group of 67 professionals, 37 (55%) responded in the first round, 38 (57%) in the second round, and 39 (58%) in the third round. Twenty-nine professionals (43%) completed all three questionnaires.

**Fig. 1:**
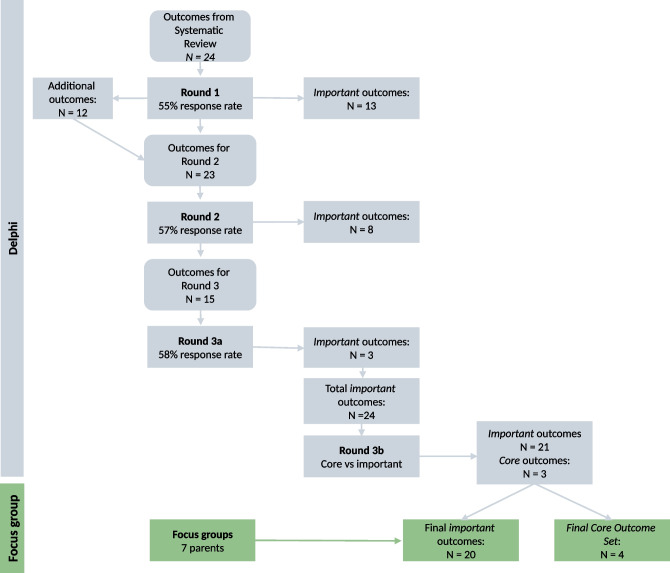
Process for development of Core Outcome Set

In round 1, consensus was reached on 13 outcomes considered important. No outcomes were excluded. The professionals suggested 12 additional outcomes. Round 2 included the 11 outcomes that remained from the first round for re-rating, along with the 12 additional suggested outcomes. Consensus was reached on eight important outcomes. Again, no outcomes were excluded. In round 3, professionals reached a consensus on three more important outcomes and no outcomes were excluded. A flowchart of the study is presented in Fig. 1. The results of the three Delphi rounds are summarized in Table 2.

**Table 2:** Results of the Delphi study

	Round 1	Round 2	Round 3a	Round 3b
	5-point Likert scale			Yes/no
*Mortality and physical health*				
*Mortality/survival of children*^a^	*81.1%*	*-*	*-*	*51.0%*
***Disease management***^a^	***70.0%***	***-***	***-***	***74.4%***
Parental physical health^b^	-	63.2%	65.8%	-
Development of the child^b^	-	60.5%	65.8%	-
*Life impact*				
***Quality of life of the children***^a^	***100.0%***	***-***	***-***	***84.6%***
*Quality of life of the parents*^a^	*94.6%*	*-*	*-*	*69.2%*
*Anxiety of the parents*^a^	*73.0%*	*-*	*-*	*25.6%*
Depression of the parents^a^	62.2%	63.2%	65.8%	-
*Self-efficacy of the parents*^a^	*91.9%*	*-*	*-*	*46.2%*
Self-efficacy of the child^b^	-	50.0%	57.9%	-
Behavior problems of the child, e.g., aggression and hyperactivity^a^	48.6%	65.8%	68.4%	-
*Satisfaction of parents with the received transitional care*^a^	*89.2%*	*-*	*-*	*41.0%*
*Satisfaction of parents with the received family-centered care*^a^	*73.0%*	*-*	*-*	*35.9%*
Satisfaction of parents with the hospital care in generally^a^	54.1%	47.4%	42.1%	-
The experiences of the child with the multidisciplinary transitional care^b^	-	55.3%	65.8%	-
*The experiences of parents with the multidisciplinary transitional care*^b^	*-*	*63.2%*	*78.9%*	*-*
*The knowledge, skills, and competencies of parents to provide the care for their children*^a^	*83.8%*	*-*	*-*	*66.7%*
*Compliance of parents, e.g., follow-up appointments*^a^	*64.9%*	*89.5%*	*-*	*48.7%*
*Compliance of children, e.g., follow-up appointments*^a^	*59.5%*	*68.4%*	*84.2%*	*-*
*Post-traumatic stress symptoms in children*^b^	*-*	*76.3%*	*-*	*28.2%*
*Post-traumatic stress symptoms in parents*^b^	*-*	*65.8%*	*81.6%*	*-*
***Impact on the life of the family***^b^	***-***	***92.1%***	***-***	***74.4%***
*Impact on the life of the child*^b^	*-*	*92.1%*	*-*	*69.2%*
*Impact on the life of siblings*^b^	*-*	*78.9%*	*-*	*30.8%*
*Resource use*				
*Hospital re-admissions*^a^	*86.5%*	*-*	*-*	*61.5%*
*PICU (re-)admissions*^a^	*73.0%*	*-*	*-*	*51.3%*
*Visits to the emergency department*^a^	*73.0%*	*-*	*-*	*48.7%*
*Hospital length of stay*^a^	*75.3%*	*-*	*-*	*48.7%*
*Number of contact moments with the outpatient department (outpatient clinic)*^a^	*67.6%*	*76.3%*	*-*	*33.3%*
*Number of primary-care consultations or visits to a community-based clinic*^a^	*59.5%*	*81.6%*	*-*	*28.2%*
Number of activities performed by primary-care professionals, e.g., laboratory tests, examinations, and coordination services^a^	35.1%	18.4%	15.8%	-
Costs of healthcare use^a^	54.1%	68.4%	63.2%	-
Inappropriate use of a facility^b^	-	47.4%	52.6%	-
*Adverse events*				
*Number and nature of errors and harm at home*^a^	*54.1%*	*81.6%*	*-*	*38.5%*
*Other*				
Staff perception about transitional care, in terms of feasibility, usability, and satisfaction^a^	56.8%	68.4%	63.2%	-
The experiences of the healthcare professionals with the multidisciplinary transitional care^b^	-	55.3%	57.9%	-

The italic outcomes represent those outcomes that reached consensus as important outcomes after the Delphi study. The bold outcomes represent those consensus that reached consensus as core outcomes after the Delphi study

^a^Based on literature

^b^Additional outcome suggested by participants in round 1

As mentioned in the “Methods” section, in the second part of round 3 a distinction was made between a *core* and *important* outcome. This resulted in three *core* outcomes: (1) disease management, (2) child’s quality of life, and (3) impact on the life of the family. See Table 2.

### Focus groups

Two online focus groups of approximately 1 h were held in March 2022. Of the 27 parents invited, nine did not respond, eight declined participation, and three did not show up on the focus group day. In total, seven parents participated in the focus groups. Characteristics of parents are presented in Supplement 1. In the focus groups, we went through all the outcomes that had already been mentioned in the information letter point by point. Parents agreed on the importance of the outcomes “child’s quality of life” and “impact on the life of the family”. The priority for the parents was the child’s comfort, and it was emphasized that the child’s well-being affected the whole family, including siblings. In addition, all parents stated that the self-efficacy of parents to provide the needed care for their child was crucial. Parents explained that even though they were trained in the hospital to care for the child, they often felt insecure without healthcare professionals nearby. Therefore, this outcome was added to the list of core outcomes.

### Final core outcome set

The final set includes four *core* outcomes: (1) disease management, (2) child’s quality of life, (3) impact on the life of families, and (4) self-efficacy of parents. The COS development process resulted in 20 additional *important* outcomes. Results are summarized in Table 3 and descriptions of the core outcomes are presented in Supplement 2.

**Table 3:** Final list of core and important outcomes

**Core Outcome Set**
*Mortality and physical health*
Disease management
*Life impact*
Quality of life of the children
Self-efficacy of the parents
Impact on the life of the family

**Important outcomes**
*Mortality and physical health*
Mortality/survival of children
*Life impact*
Quality of life of the parents
Anxiety of the parents
Satisfaction of parents with the received transitional care
Satisfaction of parents with the received family-centered care
The experiences of parents with the multidisciplinary transitional care
The knowledge, skills, and competencies of parents to provide the care for their children
Compliance of parents, e.g., follow-up appointments
Compliance of children, e.g., follow-up appointments
Post-traumatic stress symptoms in children
Post-traumatic stress symptoms in parents
Impact on the life of the child
Impact on the life of siblings
*Resource use*
Hospital re-admissions
PICU (re-)admissions
Visits to the emergency department
Hospital length of stay
Number of contact moments
Number of primary-care consultations or visits to a community-based clinic
*Adverse events*
Number and nature of errors and harm at home

## Discussion

This study resulted in a COS with four core outcomes to be used in all research programs evaluating transitional care for CMC: (1) disease management, (2) child’s quality of life, (3) impact on the life of families, and (4) self-efficacy of parents. These four outcomes underpin that attention should be paid to biomedical aspects of the child, as well as psychological and social factors of the child and its family.

### Comparison with previous research

The core outcome “disease management” is used with variability of applied outcomes, such as physical development [33], physical health needs [34], weight on standard growth curve [35], and well-controlled disease [34]. This is in line with other literature where parents prioritize their desire to support their child’s growth, weight gain, and physical development [11]. However, this outcome is reported in only 8% of studies investigating the hospital-to-home transition for CMC [22]. This might be explained by the heterogeneity of diagnoses and syndromes of CMC. In addition, disease management can be operationalized by resource use such as “hospital re-admissions” and “visits to the emergency department”.

The core outcome child’s quality of life is widely acknowledged as an important outcome in many research areas [36–39]. The outcome is also included in several pediatric COSs for chronic diseases, facilitating comparisons across studies [40–42]. Surprisingly, systematic reviews on CMC health outcomes showed that child’s quality of life is measured in very few studies [22, 43]. An explanation could be that studies are designed to acquire data with as little burden as possible for parents. Since the quality of life of CMC can only be obtained by parental proxy measures, it might not have been incorporated in studies to avoid burden for the parents. However, recently a vision paper was published, placing quality of life of CMC and their families central when developing services to provide CMC and their families a life of dignity, autonomy, and independence [44].

Consensus on the core outcome impact on the life of the family echoes prior findings showing the importance that caregivers place on the needs of not only the patient, but also the family members [9, 44–46]. The impact of a child’s critical illness and hospital admission on family members may be profound as they may experience psychosocial sequelae too [47]. Family members’ responses may, in turn, influence the outcomes of child survivors following pediatric critical illness [48, 49]. In our systematic review, only one study took the family into account [50], and one study reported on out-of-the-pocket expenditures specifically [22]. A possible explanation could be that the impact of living with a CMC on the family is more explored in qualitative studies. Furthermore, it is a broad concept and might overlap with outcomes identified as important, for example, “impact on the life of siblings” and “post-traumatic stress symptoms in parents”.

The fourth core outcome is “self-efficacy of parents”. This outcome was found in 19% of the intervention studies regarding transitional care [22]. In the studies several terms were used, such as self-confidence [51] and parents’ beliefs in their caregiving skills [52]. To avoid confusion, they were gathered under the outcome self-efficacy of parents, meaning the confidence of parents in their capabilities to manage their child’s demands adequately [51]. The importance of this outcome is congruent with the review of Peer et al. that identified self-efficacy as a key factor in determining how well parents of CMC cope with their situation [53]. Another study recommends developing and testing strategies promoting parents’ self-efficacy to maximize quality of life and improve health outcomes in CMC [54]. Lastly, qualitative studies on transitional care for CMC support the consensus on the core outcome self-efficacy of parents [11, 55].

### Core versus important outcome

During the development of this COS participants noted that all outcomes were considered important, and the difference between core and important was difficult to make. Therefore, in the last Delphi round, we explained once more the idea of a COS, and explicitly asked to choose between *important* or *core* for every outcome. This resulted in three *core* outcomes and 20 *important* outcomes as preliminary results and input for the focus groups. The number of outcomes included in a COS can vary depending on the specific condition or intervention being studied. Williamson et al. stated that the number of outcomes included in COSes for pediatric clinical trials ranged from 3 to 70, with a median of 15 outcomes per COS [27]. Although there is no consensus regarding the ideal number of core outcomes in a COS, it is very likely that fewer outcomes make the implementation more feasible. An explanation for the many important outcomes might be that transitional care is a complex, multi-faceted process that can be evaluated on many different aspects.

### Parent involvement

A strength of this study is the involvement of CMC parents in the research team and focus groups. No children were involved since their diagnoses often accompany developmental delay and intellectual disability. The importance of patients’ involvement in research is acknowledged, but not yet common practice in the COS development [56]. However, the effort must be made because the mismatch between researchers and patients in research priorities and outcome selection has proven to result in considerable research waste [57]. In this study, parents in the focus groups had a crucial contribution in the final COS, as they identified the outcome self-efficacy of parents as core which differed from the professionals.

### Limitations

Our findings should be interpreted with several caveats in mind. First, the number of healthcare professionals and parents was unbalanced. In line with other studies, we experienced challenges in obtaining a representative sample of parents [56, 58, 59]. Secondly, parents were all female with the Dutch nationality, making it impossible to consider cultural differences. Thirdly, healthcare professionals from only three high-income countries were included, limiting the COS’ generalizability. Fourthly, not all participants completed all three Delphi rounds. Although this is common in Delphi studies [60], it is uncertain to what degree this influenced the consensus procedure. Finally, in round 3 of the Delphi, consensus was found on three more outcomes: compliance of children, e.g., follow-up appointments; post-traumatic stress symptoms in parents; and the experiences of parents with the multidisciplinary transitional care. For these outcomes, participants were not asked to choose between important or core. However, parents did not mention these outcomes in the focus groups, so we decided not to include them in the final COS.

### Future directions

Further implementation of the COS would be facilitated by recommendations for feasible and validated measurement tools for each core outcome [61]. Additionally, future work should be used to address the timing of the evaluation moments for each of the core outcomes. Until then, we encourage researchers to give detailed descriptions of the used measurement tools and timing of outcome assessment. An important final step will be the broad dissemination and implementation of the COS. It is important to note that this COS is not intended to restrict researchers from measuring additional outcomes that are deemed relevant to their specific study. Furthermore, as the field continues to evolve, regular updates of COS are crucial in the future. These updates ensure that COS remain comprehensive, reflective of current knowledge, and adaptable to emerging advancements.

## Conclusion

Using well-established methods, we present the COS transitional care for CMC with four core outcomes: disease management, child’s quality of life, impact on the life of the family, and self-efficacy of parents. These core outcomes could facilitate standard reporting in future research of CMC hospital to home transition.

### Supplementary Information

Below is the link to the electronic supplementary material.

Supplementary file1 (DOCX 22 KB)

## Data Availability

The data that support the findings of this study are available from the corresponding author (JM), upon reasonable request.

## Abbreviations

CMC: Children with Medical Complexity
COS: Core Outcome Set
COMET: Core Outcome Measure in Effectiveness Trials
COS-STAR: Core Outcome Set–Standards for Reporting Statement
