# Supplementary material for: Hospital-to-home transitions for children with medical complexity: part 2—a core outcome set
Source: Eur J Pediatr. 2023 Jun 20;182(9):3833–43. doi: 10.1007/s00431-023-05049-2 (PMC10570151; doi:10.1007/s00431-023-05049-2)
Supplement: Supplementary file 1 — Supplementary file1 (DOCX 22 KB) [file 431_2023_5049_MOESM1_ESM.docx]

Supplement 1: Characteristics parents focus groups (n=7)

| **Parental characteristics** | **Total** |
| --- | --- |
| Female, n (%) | 7 (100) |
| Age parent in years, median (range) | 37 (29-45) |
| Diagnostic category child, n  Neurogenetic disorder  Gastrointestinal  Respiratory  Cardiac | 4  1  1  1 |
| Relationship status, n  Co-habitant partner  Single parent | 6  1 |
| Number of siblings, median (range) | 3 (1-6) |
| Educational level, n^1^  Low  Middle  High | 0  2  5 |
| Occupational level, n^2^  Level 1  Level 2  Level 3  Level 4 | 2  1  0  4 |

^1^ Based on the subdivisions as made by the Statistics Netherlands (htpps://www.cbs.nl)

^2^ Based upon the International Isco System for professions (http://www.ilo.org/public/english/bureau/stat/isco/).

| **Core Outcome Set** | **Description** |
| --- | --- |
| Disease management | The impact of a transitional intervention program on the number of children with well controlled disease management at home (e.g. in terms of preventable physical care needs). |
| Child’s Quality of Life | Children’s Health Related Quality of Life is a multidimensional construct that measures children’s perceived impacts of health across a range of dimensions, including physical, emotional, cognitive, and social domains. |
| Impact on the life of families | The impact on the life of the family, e.g. financial stress, job retention, participation in social activities. |
| Self-efficacy of parents | Self-efficacy of parents reflects the confidence of parents in their capabilities to manage the demands of their child adequately. Synonyms used in literature are ‘self-confidence’ and ‘parents beliefs’. |

Supplement 2: Descriptions Core Outcome Set
